# Tumor necrosis factor receptor-associated protein 1 regulates hypoxia-induced apoptosis through a mitochondria-dependent pathway mediated by cytochrome c oxidase subunit II

**DOI:** 10.1186/s41038-019-0154-3

**Published:** 2019-05-23

**Authors:** Fei Xiang, Si-yuan Ma, Yan-ling Lv, Dong-xia Zhang, Hua-pei Song, Yue-sheng Huang

**Affiliations:** Institute of Burn Research, State Key Laboratory of Trauma, Burns and Combined Injury, Southwest Hospital, Third Military Medical University (Army Medical University), Chongqing, 400038 China

**Keywords:** Cardiomyocytes, Hypoxia, Tumor necrosis factor receptor-associated protein 1, Cytochrome c oxidase subunit II, Reactive oxygen species, Apoptosis

## Abstract

**Background:**

Tumor necrosis factor receptor-associated protein 1 (TRAP1) plays a protective effect in hypoxic cardiomyocytes, but the precise mechanisms are not well clarified. The study is aimed to identify the mechanism of TRAP1 on hypoxic damage in cardiomyocytes.

**Methods:**

In this study, the effects of TRAP1 and cytochrome c oxidase subunit II (COXII) on apoptosis in hypoxia-induced cardiomyocytes were explored using overexpression and knockdown methods separately.

**Results:**

Hypoxia induced cardiomyocyte apoptosis, and TRAP1 overexpression notably inhibited apoptosis induced by hypoxia. Conversely, TRAP1 silencing promoted apoptosis in hypoxic cardiomyocytes. Further investigation revealed that the proapoptotic effects caused by the silencing of TRAP1 were prevented by COXII overexpression, whereas COXII knockdown reduced the antiapoptotic function induced by TRAP1 overexpression. Additionally, changes in the release of cytochrome c from mitochondria into the cytosol and the caspase-3 activity in the cytoplasm, as well as reactive oxygen species production, were found to be correlated with the changes in apoptosis.

**Conclusions:**

The current study uncovered that TRAP1 regulates hypoxia-induced cardiomyocyte apoptosis through a mitochondria-dependent apoptotic pathway mediated by COXII, in which reactive oxygen species presents as an important component.

**Electronic supplementary material:**

The online version of this article (10.1186/s41038-019-0154-3) contains supplementary material, which is available to authorized users.

## Background

Hypoxia, known as a state of oxygen deficiency, is sufficient to induce an impairment of function. Chronic hypoxia and the resulting oxidative stress have been shown to be involved in the development and progression of cardiovascular disorders [1, 2]. Our previous study confirmed hypoxia plays an important role in the development of myocardial damage at the early stage of burn injury [3]. However, knowledge of the specific mechanism by which hypoxia affects cardiomyocytes is still unclear. Therefore, a comprehensive understanding of the biological processes leading to myocardial damage, especially hypoxia, is required. Mitochondria are known as energy factories in cells and participate in regulating endogenous apoptosis. The integrity of the structure and function of mitochondria is a key point in maintaining the physiological function of cells. Myocardial mitochondria are subjected to various stimuli, such as hypoxia. It has been shown that destroyed mitochondria, characterized by swollen mitochondrial matrix, decreased membrane potential, or ruptured outer membrane, were always noted in damaged cardiomyocytes, leading to the open of mitochondrial permeability transition pore (mPTP), cytochrome c (Cyt c) releasing from mitochondria into the cytosol, and caspase kinase activation, followed by apoptosis [4, 5]. However, the precise mechanism of hypoxia-induced mitochondrial destruction and apoptosis is still unclear.

Tumor necrosis factor receptor-associated protein 1 (TRAP1) was identified as an interacting protein for the cytoplasmic domain of tumor necrosis factor receptor 1 (TNFR1) at first [6]. The distribution of TRAP1 was in the inner mitochondrial membrane and intermembrane space. TRAP1, which was a molecular chaperone, played an essential role in the maintenance of mitochondrial homeostasis and the function of the protein transport system [7, 8]. It was confirmed that TRAP1 overexpression inhibited hypoxia-induced decrease in mitochondrial membrane potential, decline in cell viability, and increase in cell death; conversely, the knockdown of TRAP1 aggravated the hypoxia-induced cell damage. Furthermore, we found that blocking the mPTP opening prevented the injury caused by TRAP1 knockdown [9]. However, the specific mechanism has not been well clarified.

As mentioned before, TRAP1 shows a major effect on the protection of cardiomyocytes towards hypoxia; however, whether and how TRAP1 regulates energy metabolism in hypoxic cardiomyocytes remains elusive. Our previous study further revealed that cytochrome c oxidase (COX) subunit II (COXII), a component of COX, is one of the downstream effectors that mediate the TRAP1-regulated energy generation program. Previous studies on COXII have mainly focused on electron transfer and energy generation [10]. The results from our previously published study demonstrated that the COXII-COX pathway is involved in the myocardial energy productive process regulated by TRAP1 [11]. However, the effect and mechanism of COXII on the apoptosis of hypoxic cardiomyocytes are not clear. Jancura et al. [12] reported that COXII affected the production of reactive oxygen species (ROS), an apoptosis inducer [13]. Recently, in a model of chronic obstructive pulmonary disease, it was found that the disease increased the expression of COXII and the proportion of apoptosis significantly [14]. Additionally, it was reported that high COXII expression reduced ROS production in a feedback manner [15]. These results indicate that the changes in COXII are related to apoptosis process; at least, the mechanism is not achieved by reducing the production of ATP.

The aim of the present study was to explore whether TRAP1 could protect against apoptosis in hypoxic cardiomyocytes. We revealed the antiapoptotic function of TRAP1 in cardiomyocytes under hypoxic conditions and showed that COXII was one of the downstream effectors by which TRAP1 mediates its antiapoptotic effects through a mitochondria-dependent apoptotic pathway. Our findings enriched the molecular mechanisms of apoptosis adjustment in cardiomyocytes under hypoxic conditions, offering novel insight into the prevention and treatment of hypoxia-induced myocardial damage.

## Methods

### Generation of viral constructs

The recombinant viral vectors in the present study were constructed as previously described [9, 11]. Adenovirus-mediated overexpression vectors encoding TRAP1 (Ad_TRAP1) and COXII (Ad_COXII) were produced. Adenoviral vectors expressing shRNA specifically targeting TRAP1 (TRAP1_shRNA) and COXII (COXII_shRNA), respectively, were also generated. Moreover, vectors encoding the green fluorescent protein (GFP) sequence and vectors containing nonspecific shRNA were constructed in the same way and served as negative controls correspondingly.

### Cardiomyocyte culture and the generation of a hypoxic model

Animals that were used in the present study were approved by the Animal Care and Use Committee (IACUC) of Third Military Medical University (Army Medical University). The isolation and cultivation of ventricular cardiomyocytes derived from neonatal Sprague-Dawley rats (days 1–3), as well as the generation of hypoxic cardiomyocytes, were achieved by previous methods [11]. In brief, isolated cardiomyocytes were plated on collagen-coated plates and cultured in DMEM/F12 medium (HyClone, USA) with 10% FBS (HyClone, USA), 0.1 mM bromodeoxyuridine (Sigma, USA), 100 U/ml penicillin, and 100 U/ml streptomycin (Sigma, USA) in an incubator of 5% CO_2_ at 37 °C. Hypoxic medium was produced by placing serum-free medium into a vacuum glove box filled with a mixed gas containing 94% nitrogen, 5% CO_2_, and 1% O_2_ overnight. Ultimately, a hypoxic cardiomyocyte model was generated by replacing the normal medium with hypoxic medium in an anaerobic jar for 6 h (Mitsubishi, Japan).

### Determination of apoptosis in cardiomyocytes

According to the manufacturer’s instructions, terminal deoxynucleotidyl transferase-mediated dUTP nick-end labeling (TUNEL) (Roche, Germany) was used to determine the apoptosis. Briefly, after rewarming, incubating, and washing, the cells were labeled with TUNEL reaction solution and dyed with diaminobenzidine (DAB) buffer. The images of TUNEL staining were observed with an inverted microscope, and ten fields were selected randomly to count TUNEL-positive cells. The positive cells are expressed as a percentage of total cells in the same fields, which were considered as the apoptosis rates.

### Detection of cytochrome c (Cyt c) in the cytosol and mitochondria

Cyt c levels in the cytosolic fraction and mitochondrial fraction, representing the leakage of Cyt c from mitochondria into the cytosol, were detected by western blotting assay. Mitochondrial and cytosolic fractions were extracted using the ProteoExtract® Cytosol/Mitochondria Fractionation Kit from Millipore Biotech Company. Briefly, 5 × 10^7^ cells were collected, centrifuged at 600×*g*, resuspended using 1× cytosol extraction buffer, incubated on ice, and sufficiently homogenized. When the last centrifugation ended, the supernatant was the cytosolic fraction and the pellet resuspended with mitochondria extraction was the mitochondrial fraction. The fractions were collected and used as an objective protein for western blotting.

### Western blotting analysis

The objective protein samples were separated on 12% SDS-PAGE. The separated proteins were electrotransferred to polyvinylidene fluoride (PVDF) membranes. The membranes were incubated with rat anti-Cyt c polyclonal antibodies (Abcam, USA) at 4 °C overnight after being blocked in Tris-buffered saline and Tween (TBST) with 5% skim milk for 1 h at room temperature (RT). The membrane was washed using TBST three times for 8 min each and then incubated with a goat anti-rat IgG secondary antibody (Abcam, USA) for 1 h at RT. ECL was used to reveal the protein bands, and Quantity One 4.1 software (Bio-Rad, USA) was applied to quantify the bands.

### Detection of caspase-1 and caspase-3 activities

The caspase-1 activity and caspase-3 activity in the myocyte lysates were determined using the Caspase-1 or Caspase-3 Colorimetric Assay Kit separately (BioVision, USA). According to instructions, a total of 5 × 10^6^ cells were collected, resuspended in ice-cold cell lyses buffer, incubated on ice for 10 min, and centrifuged at 10000×*g* for 1 min. The supernatant, which served as a detection sample, was collected and incubated with 2× reaction buffer (including 10 mM DTT) and 4 mM DEVD-pNA buffer for 2 h at 37 °C. A microplate reader was used to detect the absorbance at 405 nm. At least three independent experiments were performed.

### ROS detection

The release of ROS was detected using an ROS assay kit (Sigma, USA). Briefly, a 40-μl DMSO mixed with 500× ROS detection reagent was initially prepared and used as the reaction buffer. A total of 10^6^ cells from each group were obtained, resuspended in reaction buffer, and incubated in an incubator with 5% CO_2_ at 37 °C for 1 h. ROS activity was detected by flow cytometry (FCM). At least three independent experiments were performed.

### Statistical analysis

The results are expressed as the means ± SEM and analyzed by using the SPSS 21.0 statistical software (SPSS Inc., Chicago, IL, USA). Statistical analysis of multiple groups used one-way analysis of variance followed by post hoc Tukey’s tests was used for. It was considered that *p* < 0.05 was statistically significant.

## Results

### TRAP1 regulates apoptosis in hypoxic cardiomyocytes

**Fig. 1:**
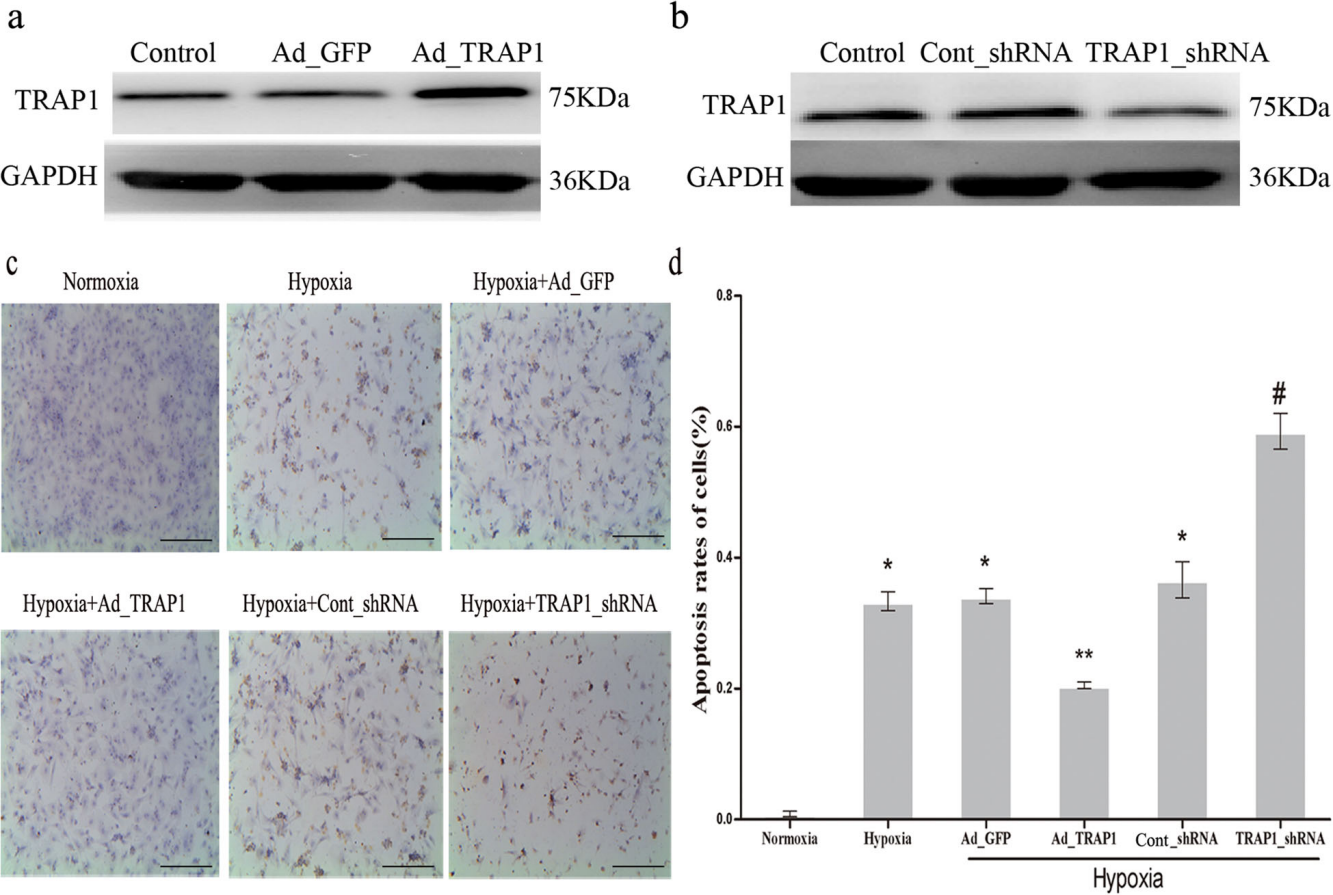
Tumor necrosis factor receptor-associated protein 1 (TRAP1) regulates apoptosis in hypoxic cardiomyocytes. **a**, **b** Confirmation of viral-mediated overexpression or knockdown of TRAP1 in cardiomyocytes. **a** Western blotting demonstrated TRAP1 overexpression mediated by adenovirus (Ad_TRAP1). Adenoviral vector encoding green fluorescent protein (Ad_GFP) served as a negative control. **b** Western blotting demonstrated TRAP1 decrease mediated by adenoviral shRNA (TRAP1_shRNA). Adenoviral-mediated expression of scrambled shRNA (Cont_shRNA) served as a negative control. **c**. Representative images of cardiomyocyte apoptosis were detected by TUNEL staining after Ad_TRAP1 or TRAP1_shRNA transfection with or without hypoxia. TUNEL staining (brown yellow) indicates apoptotic cells. Scale bar = 50 μm. **d** Apoptosis rates of cardiomyocytes were quantitatively analyzed in each treatment group. The percentage of apoptotic cells was calculated by randomly selecting ten fields. *n* = 5/group. *p < 0.05 vs normoxia group; **p < 0.05 vs hypoxia group and hypoxia+Ad_GFP group, respectively; #*p* < 0.05 vs hypoxia group and hypoxia+cont_shRNA group, respectively. The oxygen concentration is 1%. Data are presented as mean ± SEM. Error bars indicate SEM, *p* value was analyzed using post hoc Tukey’s tests. The experiment was repeated three times. *GAPDH* glyceraldehyde-3-phosphate dehydrogenase, *TUNEL* terminal deoxynucleotidyl transferase-mediated dUTP nick-end labeling

Overexpression and knockdown approaches were applied to explore the correlation between TRAP1 expression and apoptosis in hypoxia-induced cardiomyocytes. Adenoviral vectors that encoded GFP as a control (Ad_GFP) or TRAP1 (Ad_TRAP1) were generated at first. The western blotting results revealed that TRAP1 expression increased significantly in the Ad_TRAP1 group than those in the Ad_GFP group and in the control group (Fig. 1a). Then, we also generated adenoviral-mediated expression of scrambled shRNA (Cont_shRNA) or shRNA specifically targeting TRAP1 (TRAP1_shRNA), and both had a GFP tag. Western blotting was also used to confirm the effective silencing of endogenousTRAP1 by TRAP1_siRNA adenovirus infection (Fig. 1b). Then, a TUNEL assay was performed to observe the morphological characteristics of apoptosis in cardiomyocytes. Six treatment groups were divided randomly: normoxia, hypoxia, hypoxia+Ad_GFP, hypoxia+Ad_TRAP1, hypoxia+Cont_shRNA, and hypoxia+TRAP1_shRNA. Besides the normoxia group, cardiomyocytes in the other groups were cultured under hypoxic conditions for 6 h. As shown in Fig. 1c, hypoxia induced obvious morphological characteristics of cardiomyocyte apoptosis. The overexpression of TRAP1 significantly reduced cardiomyocyte apoptosis, while TRAP1 silencing showed the opposite effect (Fig. 1c, d) in response to hypoxia. However, since pyroptosis can also induce a TUNEL-positive assay, we further determined whether TRAP1 have a role on pyroptosis. As caspase-1 is a key mediator in pyroptosis process, the effect of TRAP1 on caspase-1 activity was tested both in normoxic and hypoxic cardiomyocytes. The results showed TRAP1 had no effect on caspase-1 activity both in normoxic and hypoxic cardiomyocytes, and caspase-1 activities between hypoxia group and normoxia group had no significant differences (Additional file 1: Figure S1). As a result, the data indicated that TRAP1 plays an antiapoptotic role in hypoxic cardiomyocytes.

### TRAP1 regulates the release of apoptosis-associated factors in hypoxic cardiomyocytes

**Fig. 2:**
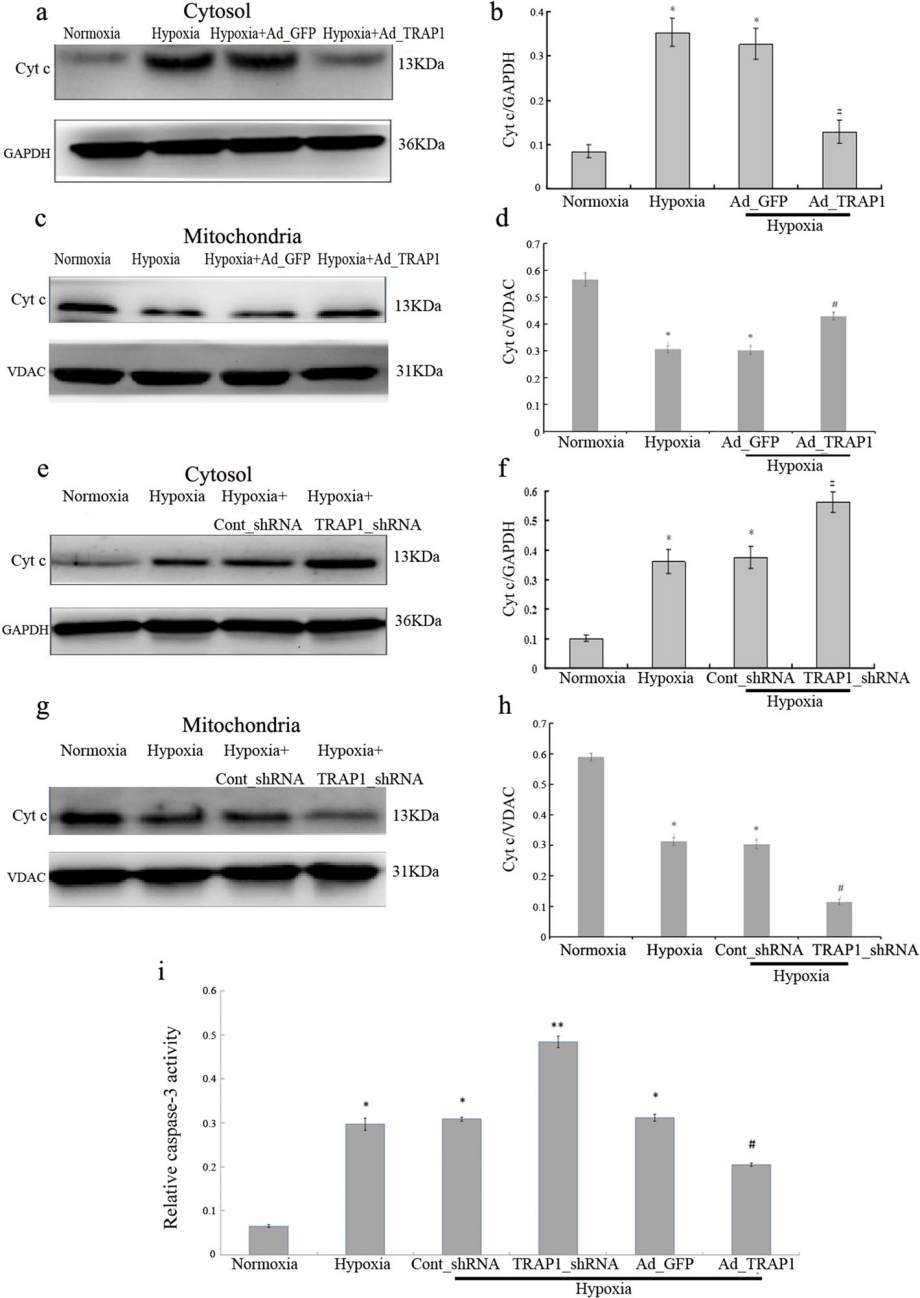
Tumor necrosis factor receptor-associated protein 1 (TRAP1) regulates the release of apoptosis-associated factors in hypoxic cardiomyocytes. **a**–**d** Western blotting and quantitative analysis were used to show the expression of cytochrome c (Cyt c) in the cytosol (**a**, **b**) and in the mitochondria (**c**, **d**) after Ad_GFP or Ad_TRAP1 transfection with or without hypoxia, indicating the release of Cyt c from mitochondria into the cytosol. *p < 0.05 vs normoxia group; # *p* < 0.05 vs hypoxia group and hypoxia+Ad_GFP group, respectively. **e**–**h** Western blotting and quantitative analysis were used to show the expression of Cyt c in the cytosol (**e**, **f**) and in the mitochondria (**g**, **h**) after Cont_shRNA or TRAP1_shRNA transfection with or without hypoxia. *p < 0.05 vs normoxia group; #*p* < 0.05 vs hypoxia group and hypoxia+cont_shRNA group, respectively. **i** Caspase-3 activity in the myocyte lysates was determined using the caspase-3 Colorimetric Assay Kit after Ad_TRAP1 or TRAP1_shRNA transfection with or without hypoxia. *p < 0.05 vs normoxia group; **p < 0.05 vs hypoxia group and hypoxia+cont_shRNA group, respectively; #*p* < 0.05 vs hypoxia group and hypoxia+Ad_GFP group, respectively. Data are presented as mean ± SEM. Error bars indicate SEM, *p* value was analyzed using post hoc Tukey’s tests. The experiment was repeated three times. *Ad_TRAP1* adenoviral vector encoding TRAP1, *Ad_GFP* adenoviral vector encoding green fluorescent protein, *TRAP1_shRNA* adenoviral shRNA specifically targeting TRAP1, *Cont_shRNA* adenoviral-mediated expression of scrambled shRNA,* GAPDH* glyceraldehyde-3-phosphate dehydrogenase

To determine whether TRAP1 had any effects on the apoptosis of cardiomyocytes under hypoxic conditions, mitochondrial Cyt c leakage was first detected. The grouping and cultivation of cardiomyocytes were performed as mentioned above. The expression level of Cyt c in the cytosol in the hypoxia group was significantly increased than that in the normoxia group, and overexpression of TRAP1 (Ad_TRAP1) remarkably inhibited the hypoxia-induced increase in Cyt c detected in the cytosol (Fig. 2a, b). Meanwhile, the Cyt c expression level in the hypoxia+Ad_TRAP1 group was notably increased compared to that in the hypoxia group in the mitochondria (Fig. 2c, d). Moreover, we further investigated the effect of TRAP1 (TRAP1_shRNA) knockdown on Cyt c in the cytosol and in the mitochondria. The results showed the Cyt c level in the hypoxia+TRAP1_shRNA group was significantly increased in the cytosolic fraction (Fig. 2e, f) and decreased in the mitochondrial fraction than that in the hypoxia group (Fig. 2g, h), indicating TRAP1 expression protected cardiomyocytes against apoptosis induced by hypoxia. Furthermore, caspase-3 activity was detected and the activity in the hypoxia group was robustly increased compared to that in the normoxia group, whereas little difference was noted between the hypoxia group and the hypoxia+Ad_GFP group. However, TRAP1 overexpression dramatically suppressed the elevated caspase-3 activity in cardiomyocytes under hypoxic conditions. Conversely, the knockdown of TRAP1 (TRAP_shRNA) significantly raised the caspase-3 activity when compared with the hypoxia group (Fig. 2i). Taken together, these findings demonstrated that TRAP1 protects cardiomyocytes from hypoxia-induced apoptosis.

### COXII knockdown prevents the antiapoptotic effect of Ad_TRAP1 on hypoxic cardiomyocytes

**Fig. 3:**
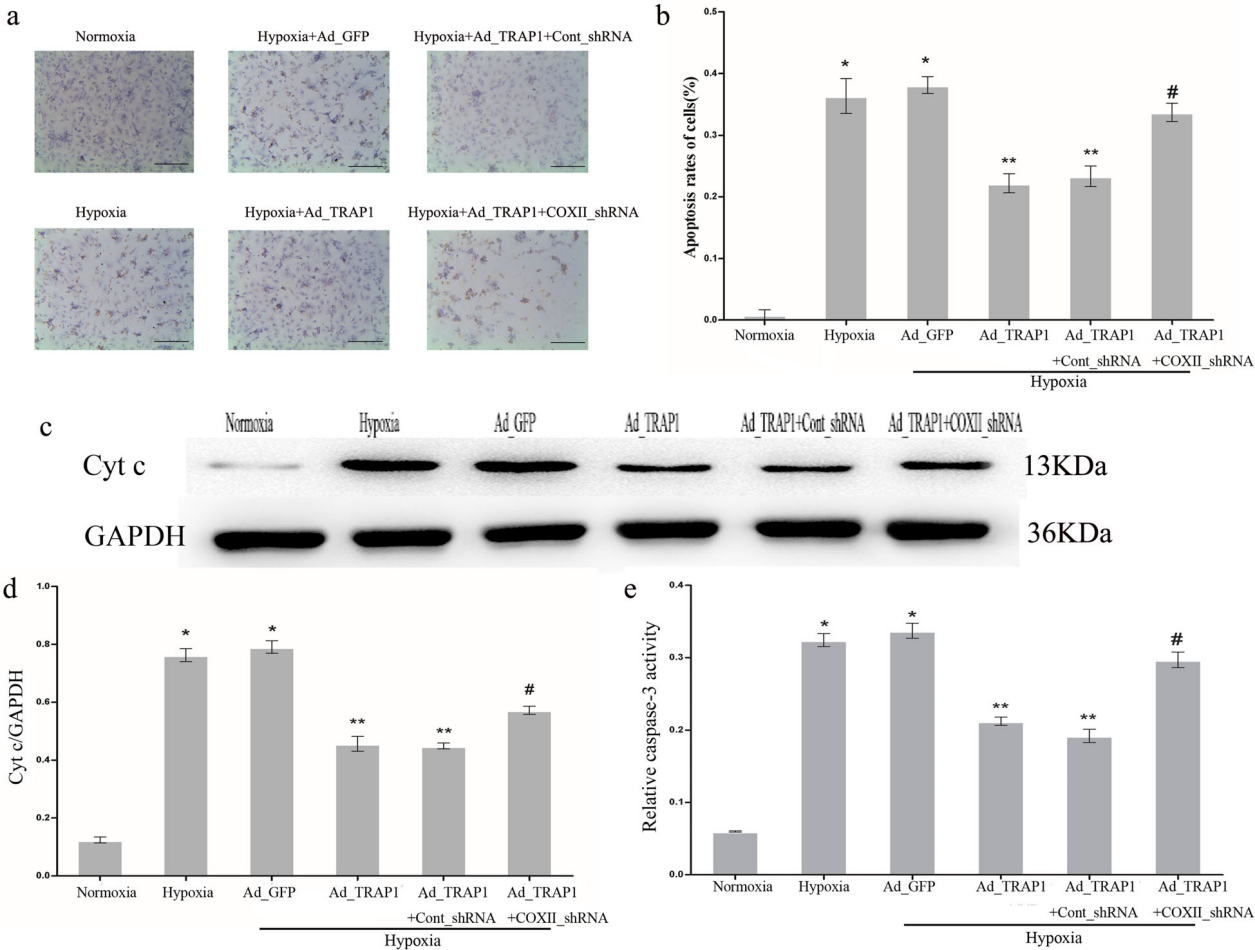
Cytochrome c oxidase subunit II (COXII) knockdown prevented the antiapoptotic effect of Ad_TRAP1 on hypoxic cardiomyocytes. **a** Representative images of cardiomyocyte apoptosis were detected by TUNEL staining after transfection with Ad_TRAP1, COXII_shRNA, or both, with or without hypoxia. Scale bar = 50µm. **b** Apoptosis rates of cardiomyocytes were quantitatively analyzed in each treatment group. **c**, **d** Western blotting (**c**) and quantitative analysis (**d**) were performed to detect cytochrome c (Cyt c) levels in the cytosol. GAPDH served as an internal control. **e** Caspase-3 activity in the cardiomyocytes was determined using the caspase-3 Colorimetric Assay Kit in each treatment group. *p < 0.05 vs normoxia group; **p < 0.05 vs. hypoxia group and hypoxia+Ad_GFP group, respectively; #*p* < 0.05 vs hypoxia+Ad_TRAP1 group and hypoxia+Ad_TRAP1+Cont_shRNA group, respectively. Data are presented as mean ± SEM. Error bars indicate SEM, *p* value was analyzed using post hoc Tukey’s tests. The experiment was repeated three times. *Ad_TRAP1* adenoviral vector encoding TRAP1, *Ad_GFP* adenoviral vector encoding green fluorescent protein, *COXII_shRNA* adenoviral shRNA specifically targeting COXII, *Cont_shRNA* adenoviral-mediated expression of scrambled shRNA, *GAPDH* glyceraldehyde-3-phosphate dehydrogenase, *TUNEL* terminal deoxynucleotidyl transferase-mediated dUTP nick-end labeling

Our previous study revealed that COXII is one of the downstream effectors involved in the TRAP1-mediated energy generation program [11]. However, whether COXII participates in the TRAP1-regulated apoptotic process in hypoxic cardiomyocytes remains unknown. To explore the effect of COXII on apoptosis, cardiomyocytes were transfected with COXII_shRNA or Cont_shRNA. The morphological characteristics of apoptosis were determined by TUNEL assay. The cells were separated into normoxia group, hypoxia group, hypoxia+Ad_GFP group, hypoxia+Ad_TRAP1 group, hypoxia+Ad_TRAP1+Cont_shRNA group, and hypoxia+Ad_TRAP1+COXII_shRNA group. The results of TUNEL analysis (Fig. 3a, b) showed that the apoptotic rate of the hypoxic cardiomyocytes in the hypoxia+Ad_TRAP1+COXII_shRNA group was higher than that in the hypoxia+Ad_TRAP1 group. In addition, a significant increase in the release of mitochondrial Cyt c and the activity of caspase-3 was detected in the hypoxia+Ad_TRAP1+COXII_shRNA group compared with the hypoxia+Ad_TRAP1 group (Fig. 3c, e). Collectively, these findings indicate that COXII knockdown mediated by COXII_shRNA partially prevented the antiapoptotic effect of Ad_TRAP1 on cardiomyocytes under hypoxic conditions.

### COXII overexpression prevented the proapoptotic effect of TRAP1_shRNA on hypoxic cardiomyocytes

**Fig. 4:**
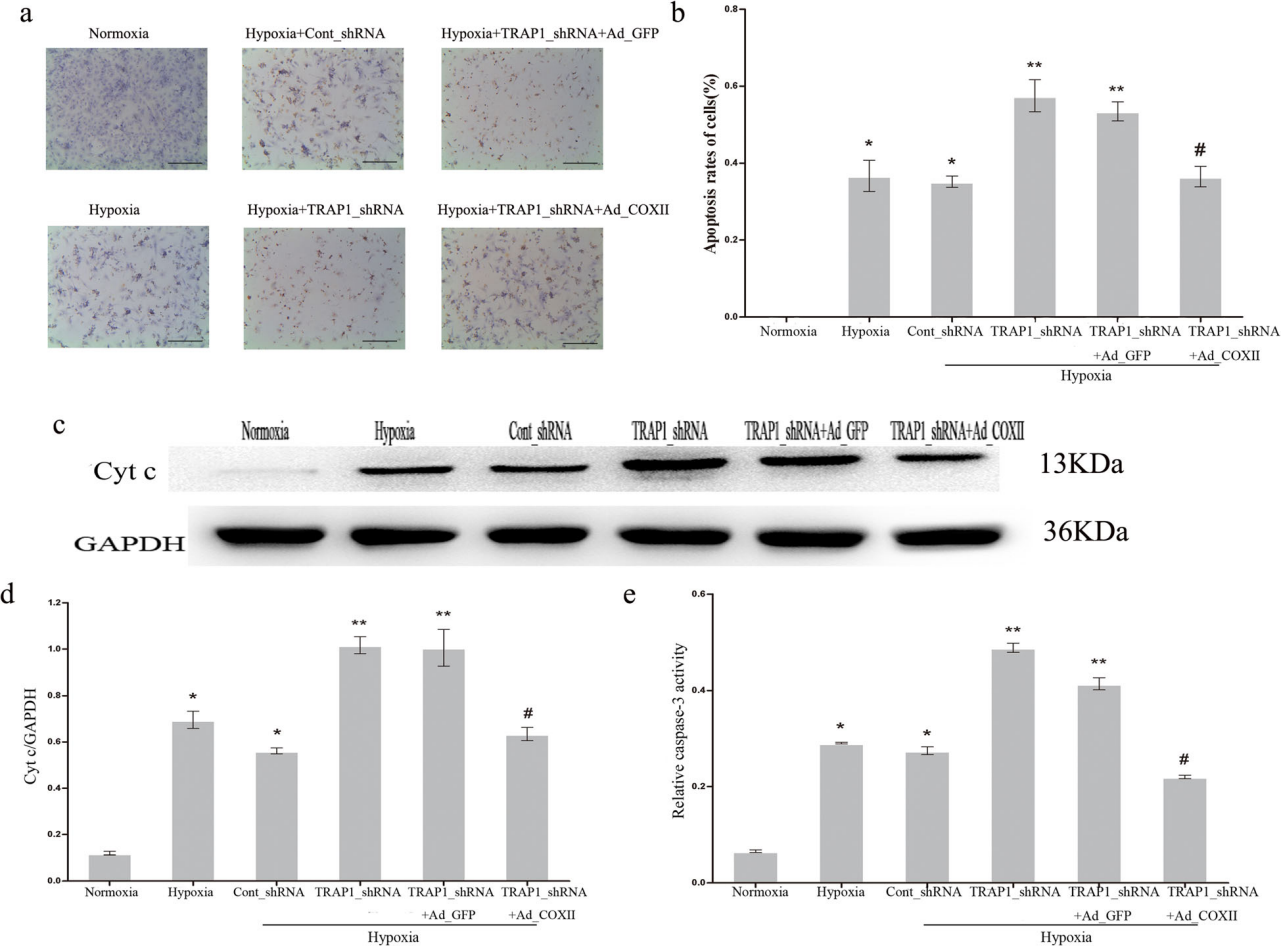
Cytochrome c oxidase subunit II (COXII) overexpression attenuates the proapoptotic effect of TRAP1_shRNA on hypoxic cardiomyocytes. **a** Representative images of cardiomyocyte apoptosis were detected by TUNEL staining after transfection with TRAP1_shRNA, Ad_ COXII, or both, with or without hypoxia. Scale bar = 50µm. **b** Apoptotic rates of cardiomyocytes were quantitatively analyzed in each treatment group. **c**, **d** Western blotting (**c**) and quantitative analysis (**d**) were performed to detect cytochrome c (Cyt c) levels in the cytosol. GAPDH served as an internal control. **e** Caspase-3 activity in the cardiomyocytes was determined using the caspase-3 Colorimetric Assay Kit in each treatment group. *p < 0.05 vs normoxia group; **p < 0.05 vs hypoxia group and hypoxia+Cont_shRNA group, respectively; #*p* < 0.05 vs hypoxia+TRAP1_shRNA group and hypoxia+TRAP1_shRNA+Ad_GFP group, respectively. Data are presented as mean ± SEM. Error bars indicate SEM, *p* value was analyzed using post hoc Tukey’s tests. The experiment was repeated three times. *Ad_COXII* adenoviral vector encoding COXII, *Ad_GFP* adenoviral vector encoding green fluorescent protein, *TRAP1_shRNA* adenoviral shRNA specifically targeting TRAP1, *Cont_shRNA* adenoviral-mediated expression of scrambled shRNA, *GAPDH* glyceraldehyde-3-phosphate dehydrogenase, *TUNEL *terminal deoxynucleotidyl transferase-mediated dUTP nick-end labeling

To further demonstrate that COXII is the key molecule of TRAP1-regulated apoptosis in hypoxic cardiomyocytes, an adenoviral-mediated overexpressing vector encoding COXII was employed. The cells were divided into six groups randomly: normoxia, hypoxia, hypoxia+Cont_shRNA, hypoxia+TRAP1_shRNA, hypoxia+TRAP1_shRNA+Ad_GFP, and hypoxia+TRAP1_shRNA+Ad_COXII. We observed a decreased cardiomyocyte apoptotic rate in the hypoxia+TRAP1_shRNA+Ad_COXII group compared to the hypoxia+TRAP1_shRNA group (Fig. 4a, b). The knockdown of TRAP1 increased the leakage of mitochondrial Cyt c in hypoxic cardiomyocytes, which was not noted in Cont_shRNA-transfected cells. The overexpression of COXII substantially reduced this increase (Fig. 4c, d). Additionally, as shown in Fig. 4e, the change in caspase-3 activity was consistent with that noted in Cyt c detection. Thus, all the findings demonstrated that COXII overexpression partially attenuated the proapoptotic effect of TRAP1_shRNA on cardiomyocytes under hypoxic conditions, supporting the premise that COXII took part in the TRAP1-regulated apoptotic process in hypoxic cardiomyocytes.

### Measurement of ROS

**Fig. 5:**
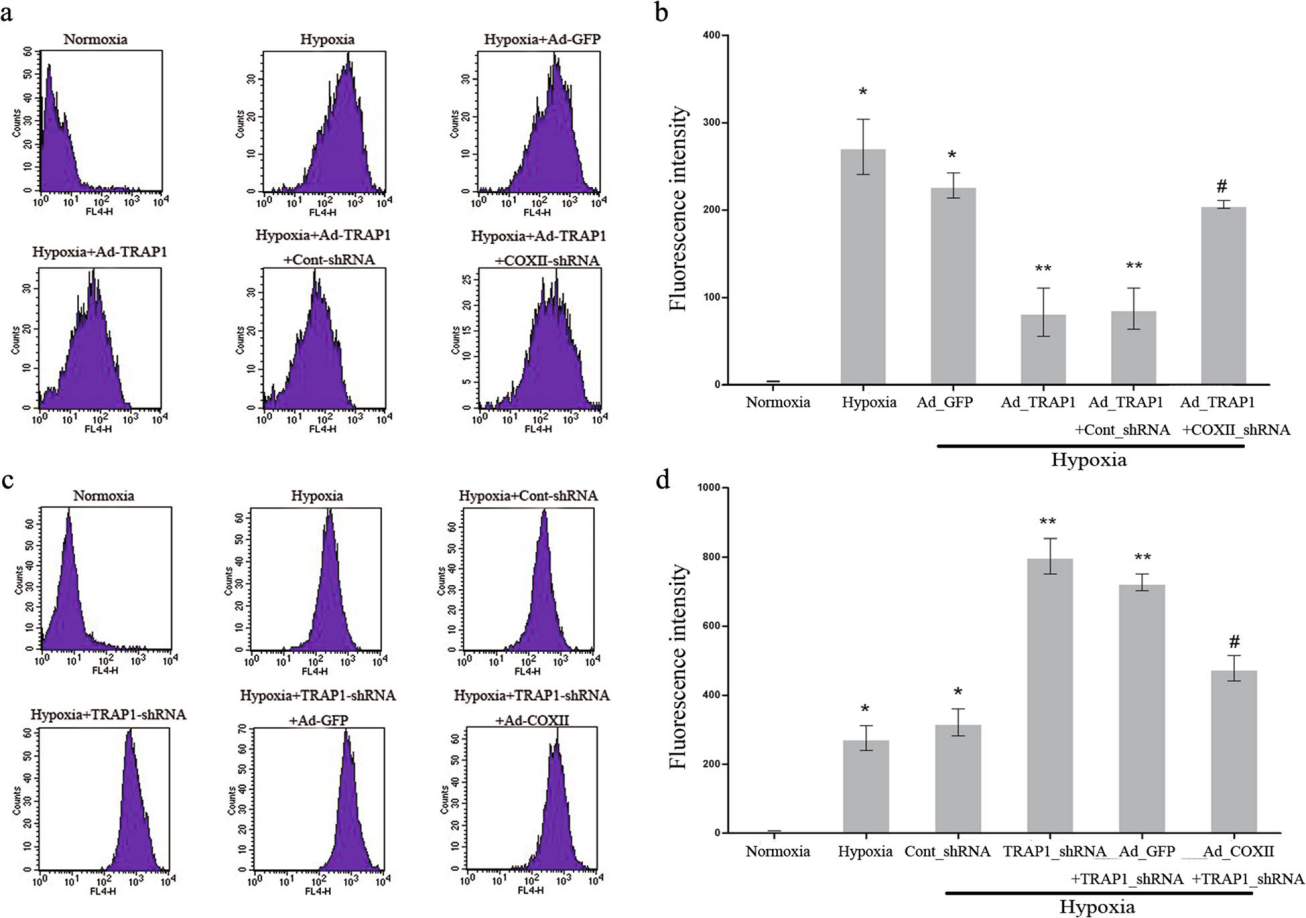
Measurement of reactive oxygen species (ROS). **a**, **b** Representative images of flow cytometry (**a**) and quantitative analysis (**b**) with respect to ROS release in hypoxic cardiomyocytes by transfection with Ad_TRAP1, COXII_shRNA, or both. *n* = 3/group. *p < 0.05 vs normoxia group; **p < 0.05 vs hypoxia group and hypoxia+Ad_GFP group, respectively; #*p* < 0.05 vs hypoxia+Ad_TRAP1 group and hypoxia+Ad_TRAP1+Cont_shRNA group. **c**, **d** Representative images of flow cytometry (**c**) and quantitative analysis (**d**) of ROS release in hypoxic cardiomyocytes by transfection with TRAP1_shRNA, Ad_COXII, or both. *n* = 3/group. *p < 0.05 vs normoxia group; **p < 0.05 vs hypoxia group and hypoxia+Cont_shRNA group, respectively; #*p* < 0.05 vs hypoxia+TRAP1_shRNA group and hypoxia+TRAP1_shRNA+Ad_GFP group, respectively. Data are presented as mean ± SEM. Error bars indicate SEM, *p* value was analyzed using post hoc Tukey’s tests. The experiment was repeated three times. *Ad_TRAP1* adenoviral vector encoding TRAP1, *Ad_COXII* adenoviral vector encoding COXII, *Ad_GFP* adenoviral vector encoding green fluorescent protein, *TRAP1_shRNA* adenoviral shRNA specifically targeting TRAP1, *COXII_shRNA* adenoviral shRNA specifically targeting COXII, *Cont_shRNA* adenoviral-mediated expression of scrambled shRNA

The measurement of ROS levels was acquired according to the abovementioned detection method. In accordance with the observation in Fig. 1a, the overexpression of TRAP1 obviously decreased ROS levels in hypoxic cardiomyocytes; however, COXII knockdown substantially increased this decline (Fig. 5a, b). Conversely, in line with the observation in Fig. 1c, COXII overexpression remarkably inhibited the increase in ROS level in hypoxic cardiomyocytes induced by TRAP1 silencing (Fig. 5c, d). These results revealed that ROS levels could be mediated by the TRAP1-regulated pathway via COXII, indicating that ROS contribute to the mechanism by which TRAP1 regulates apoptosis in hypoxic cardiomyocytes through COXII.

### Scavenging ROS by *N*-acetylcysteine (NAC) decreases the hypoxia-induced proapoptotic effect in cardiomyocytes

**Fig. 6:**
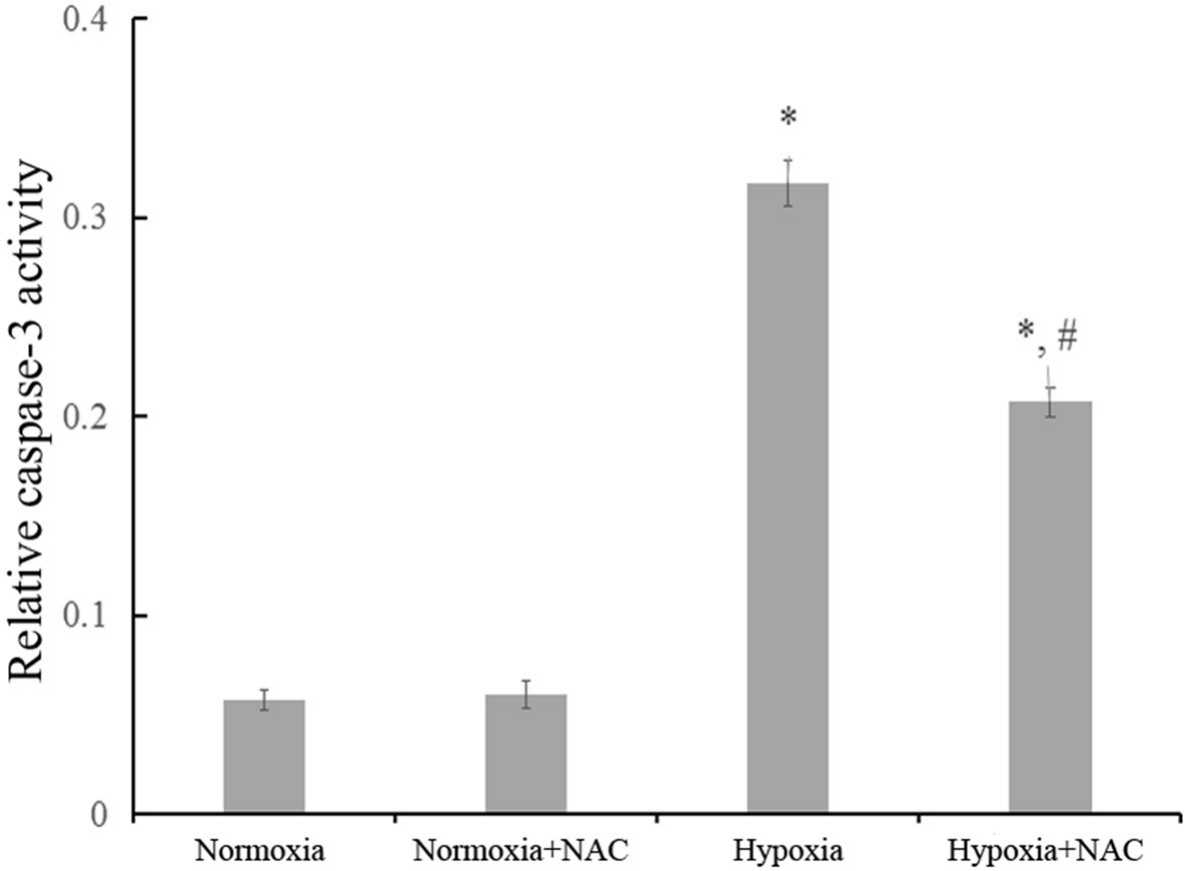
Scavenging reactive oxygen species (ROS) by *N*-acetylcysteine (NAC) decreases the proapoptotic effect induced by hypoxia in cardiomyocytes. Caspase-3 activity in the cardiomyocytes was determined in four treatment groups. Hypoxia+NAC group were pretreated with NAC (2.5 mM) for 30 min before exposure to hypoxia. *p < 0.05 vs normoxia group and normoxia+NAC group, respectively; # *p* < 0.05 vs hypoxia group. Data are the mean ± SEM. Error bars indicate SEM, *p* value was analyzed using post hoc Tukey’s tests. The experiment was repeated three times

NAC (a ROS scavenger) was used to further determine whether ROS mediates hypoxia proapoptotic effect in cardiomyocytes. The cardiomyocytes were divided into four groups: normoxia group, normoxia+NAC group, hypoxia group, and hypoxia+NAC group. Before cardiomyocytes underwent hypoxia, NAC (2.5 mM) was added and pretreated for 30 min. After the hypoxia procedure was completed, the caspase-3 activity was measured. As shown in Fig. 6, pretreatment with NAC significantly suppressed the caspase-3 activity increase in hypoxic cardiomyocytes (^#^*p* < 0.05 vs. hypoxia group), while in normoxic cardiomyocyts, NAC has no significant effect on caspase-3 activity. As a result, we demonstrated that ROS plays an important role in the proapoptotic effect induced by hypoxia in cardiomyocytes.

## Discussion

Of relevance to our topic, there is a growing awareness that chronic hypoxia is a major risk factor for the development of a variety of diseases, including cardiovascular disorders, burn injury, chronic renal insufficiency, and central nervous system diseases [16–18]. Correspondingly, the oxidative stress that occurs during intermittent or continuous cellular hypoxia is involved in all these diseases to different extents. Neurocytes in oxygen-poor conditions lead to fatal consequences. In addition, myocardial damage caused by oxygen deficits should not be underestimated because hypoxia can directly lead to myocardial injuries, resulting in myocardial dysfunction and hemodynamic disorders. Therefore, it is critical to protect cardiomyocytes against hypoxia-induced damage [19]. Understanding the underlying mechanisms of hypoxia-triggered myocardial damage not only contributes to the exploration of new therapeutic targets but also helps to develop novel methods for treatment. To date, more and more studies have been performed, and the mechanism of damage in hypoxic cardiomyocytes has been analyzed, but the results are not systematic and all-sided [20, 21]. Hypoxia is a major risk factor for apoptosis [22–24]. Maintaining the production of ATP and modulating apoptosis are two major functions of mitochondria. As a consequence, mitochondria play a key role in the response to hypoxia.

Previous studies regarding TRAP1 were mainly focused on tumor cells, showing that TRAP1 stays at the crossroad of multiple crucial processes in the initiation of neoplastic transformation and oncogenesis [25–27]. On the one hand, using an apoptotic inducer significantly inhibits the expression of TRAP1. On the other hand, TRAP1 knockdown in tumor cells induces Cyt c releasing from mitochondria into the cytosol and promotes apoptosis, whereas TRAP1 overexpression markedly attenuates the damage induced by oxidative stress and preserves the function of tumor cells [28, 29]. Although the differential expression of TRAP1 in cancer tissues plays an inhibitory role in mitochondrial apoptosis, knowledge of TRAP1 in normal tissues has not been well investigated. The results from our previous study demonstrated that TRAP1 improves the hypoxia-impaired energy production in cardiomyocytes. Hence, TRAP1 could regulate the death of normal cells in addition to tumor cells. To reveal the effect of TRAP1 on apoptosis in hypoxic cardiomyocytes, three different assays were performed to detect the percentage of apoptosis, the Cyt c levels and the caspase-3 activity. Moreover, the effect of TRAP1 on pyroptosis was also determined and the results showed pyroptosis did not activated and might not influence the downstream apoptosis factor in the present study. As reported before, mitochondrial Cyt c is an important apoptosis-inducing molecule in cells [30]. Caspase-3 presents as one of the essential executive molecules of the apoptotic process [31]. Activation of the two abovementioned molecules represents the mitochondria-dependent apoptotic pathway was activated. Mechanistically, following the activation of the apoptotic pathway, a cascade reaction was subsequently stimulated, including BCL-2 inhibition, BAX and BAK oligomerization, mitochondrial membrane permeabilization, and the release of second mitochondria-derived activator of caspase (SMAC) and Cyt c. Cyt c forms a complex with apoptotic protease-activating factor 1 (APAF-1) and procaspase-9 to promote the conversion of procaspase-9 to active caspase-9; activated caspase-9 then cascades down to the executioner caspase (caspase-3, caspase-6, and caspase-7), finally leading to apoptosis [32]. In this study, the overexpression of TRAP1 was found to play an inhibitory role in the apoptotic rate, mitochondrial Cyt c leakage, and caspase-3 activity in hypoxic cardiomyocytes, whereas the results in TRAP1 knockdown treatment were counterproductive, indicating that TRAP1 participates in the regulation of apoptosis in hypoxic cardiomyocytes in addition to its effect on energy production and that the effect of TRAP1 on apoptosis is achieved through a mitochondria-dependent apoptotic pathway. Previous study has showed TRAP1 can regulate COXII expression in cardiomyocytes [11]. Further studies revealed that the overexpression of COXII weakened the proapoptotic effect of TRAP1_shRNA; in contrast, interfering COXII expression weakened the antiapoptotic effect of Ad_TRAP1 on cardiomyocytes in response to hypoxia, indicating that COXII was involved in the TRAP1-regulated apoptotic process, that is, TRAP1 regulates the apoptosis of hypoxic cardiomyocytes via the mitochondrial-dependent apoptosis pathway mediated by COXII.

Mitochondria are the most important organelles in cardiomyocytes and are essential sites of ROS production. ROS are important apoptosis-inducing molecules [33]. A large amount of ROS will be produced in myocardial mitochondria along with the oxidative stress disequilibrium caused by hypoxia, leading to the lipid peroxidation of mitochondrial and cellular membranes, mitochondrial DNA damage, and the induction of apoptosis [34, 35]. In the present study, the correlation between ROS and TRAP1 as well as COXII was first uncovered in cardiomyocytes exposed to hypoxia. The findings showed that TRAP1 overexpression markedly decreased ROS levels in hypoxic cardiomyocytes, whereas this inhibitory effect was substantially prevented by COXII knockdown. In contrast, TRAP1 knockdown markedly increased the release of ROS in hypoxic cardiomyocytes, but this enhancement effect was attenuated by COXII overexpression. We further observed that the tendency of ROS release was consistent with the change in Cyt c release and caspase-3 activity. These findings revealed that the release of ROS directly reflected the regulatory effect of TRAP1 on apoptosis in hypoxic cardiomyocytes through the mitochondria-dependent apoptotic pathway mediated by COXII, indicating that ROS are important molecules that contribute to the modulation of TRAP1 on apoptosis through the mitochondria-dependent apoptotic pathway mediated by COXII.

## Conclusions

The present study demonstrates that TRAP1 regulates hypoxia-induced apoptosis in cardiomyocytes via the mitochondria-dependent apoptotic pathway mediated by COXII, in which ROS are important components. The molecular mechanisms underlying the hypothesized connection between TRAP1 and mitochondrial disturbances in hypoxic cardiomyocytes still require further investigation.

## Additional file

Additional file 1: **Figure S1.** Detection of caspase-1 activity. Caspase-1 activity in the cardiomyocytes by transfection with Ad_TRAP1 or TRAP1-shRNA was determined under normoxic and hypoxic conditions. Control cardiomyocytes underwent normoxia or hypoxia only. Data are presented as mean ± SEM. Error bars indicate SEM, *p* value was analyzed using post hoc Tukey’s tests. The experiment was repeated three times. *Ad_TRAP1* adenoviral vector encoding TRAP1, *Ad_GFP* adenoviral vector encoding green fluorescent protein, *TRAP1_shRNA* adenoviral shRNA specifically targeting TRAP1, *Cont_shRNA* adenoviral-mediated expression of scrambled shRNA. (TIF 1886 kb)

## Abbreviations

COXII: Cytochrome c oxidase subunit II
Cyt c: Cytochrome c
ROS: Reactive oxygen species
TRAP1: Tumor necrosis factor receptor-associated protein 1
