# Supplementary figures and images for: Tumor necrosis factor receptor-associated protein 1 regulates hypoxia-induced apoptosis through a mitochondria-dependent pathway mediated by cytochrome c oxidase subunit II
Source: Burns Trauma. 2019 May 23;7:16. doi: 10.1186/s41038-019-0154-3 (PMC6532166; doi:10.1186/s41038-019-0154-3)

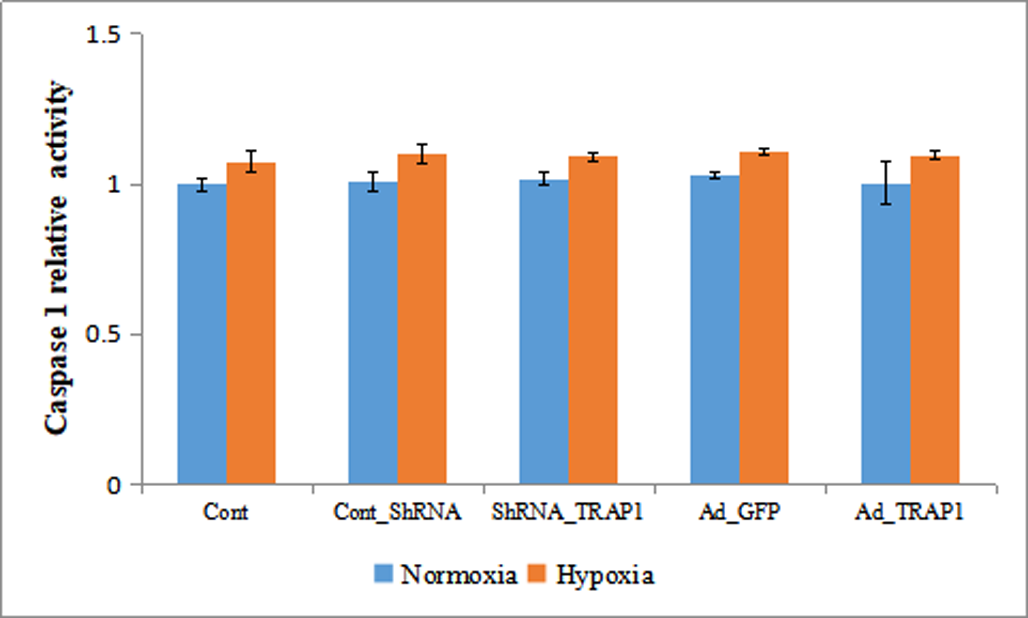

Supplement: Supplementary file 1 — Figure S1. Detection of caspase-1 activity. Caspase-1 activity in the cardiomyocytes by transfection with Ad_TRAP1 or TRAP1-shRNA was determined under normoxic and hypoxic conditions. Control cardiomyocytes underwent normoxia or hypoxia only. Data are presented as mean ± SEM. Error bars indicate SEM, p value was analyzed using post hoc Tukey’s tests. The experiment was repeated three times. Ad_TRAP1 adenoviral vector encoding TRAP1, Ad_GFP adenoviral vector encoding green fluorescent protein, TRAP1_shRNA adenoviral shRNA specifically targeting TRAP1, Cont_shRNA adenoviral-mediated expression of scrambled shRNA. (TIF 1886 kb) [file 41038_2019_154_MOESM1_ESM.tif]
